# Adversarial Evasion Attacks on SVM-Based GPS Spoofing Detection Systems

**DOI:** 10.3390/s25196062

**Published:** 2025-10-02

**Authors:** Sunghyeon An, Dong Joon Jang, Eun-Kyu Lee

**Affiliations:** Department of Information and Telecommunication Engineering, Incheon National University, Incheon 22012, Republic of Korea; asd4314@inu.ac.kr (S.A.); waitnomore@inu.ac.kr (D.J.J.)

**Keywords:** connected autonomous vehicle, GPS spoofing, spoofing detection, support vector machine, adversarial machine learning, security, attack

## Abstract

GPS spoofing remains a critical threat in the use of autonomous vehicles. Machine-learning-based detection systems, particularly support vector machines (SVMs), demonstrate high accuracy in their defense against conventional spoofing attacks. However, their robustness against intelligent adversaries remains largely unexplored. In this work, we reveal a critical vulnerability in an SVM-based GPS spoofing detection model by analyzing its decision boundary. Exploiting this weakness, we introduce novel evasion strategies that craft adversarial GPS signals to evade the SVM detector: a data location shift attack and a similarity-based noise attack, along with their combination. Extensive simulations in the CARLA environment demonstrate that a modest positional shift reduces detection accuracy from 99.9% to 20.4%, whereas similarity to genuine GPS noise-driven perturbations remain largely undetected, while gradually degrading performance. A critical threshold reveals a nonlinear cancellation effect between similarity and shift, underscoring a fundamental detectability–impact trade-off. To our knowledge, these findings represent the first demonstration of such an evasion attack against SVM-based GPS spoofing defenses, suggesting a need to improve the adversarial robustness of machine-learning-based spoofing detection in vehicular systems.

## 1. Introduction

Autonomous vehicles (AVs) and other cyber–physical systems heavily rely on the Global Positioning System (GPS) for navigation and safety-critical functions. However, GPS signals are vulnerable to malicious interference. In particular, GPS *spoofing* attacks—where an attacker broadcasts counterfeit GPS signals to deceive a receiver—pose a serious threat to positioning and timing systems. Such attacks can mislead vehicles into incorrect navigation decisions, with potentially dangerous consequences. A well-known incident in 2011 illustrated this danger: a military drone was reportedly hijacked by spoofed GPS signals and forced to land in an unintended location [1]. Ensuring the security and integrity of GPS information is therefore paramount for the safe operation of autonomous and connected vehicles.

In response to these threats, researchers have actively explored techniques for detecting GPS spoofing. Traditional approaches have used consistency checks and signal analysis, while more recent studies leveraged machine learning (ML) algorithms to distinguish genuine vs. fake GPS measurements [2]. Support vector machines (SVMs) have notably been applied as effective classifiers for GPS spoofing detection. For example, Filippou et al. developed a data-driven ML scheme to detect GPS spoofing in autonomous vehicles [3], and Kamal et al. proposed an SVM-based detection method to enhance vehicular navigation security [4]. In the domain of unmanned aerial vehicles (UAVs), Panice et al. demonstrated that an SVM classifier could successfully identify spoofing attacks on a drone’s GPS data under simulated conditions [5]. Many of these SVM-based detectors report high accuracy; for instance, supervised learning models (including SVMs) have achieved over 99% detection accuracy on benchmark spoofing datasets [6] and, in some evaluations, SVMs were shown to outperform other classifiers even in varied spoofing scenarios ranging from simplistic to more sophisticated attacks [2]. These results suggest that SVMs are a promising approach for GPS spoofing detection.

However, despite the progress made in developing detection techniques, important gaps remain in our understanding of their robustness. Most existing studies have demonstrated performance using standard scenarios or fixed datasets (e.g., the TEXBAT GNSS spoofing dataset) [7] and focused on improving overall detection rates; meanwhile, little attention has been given to the potential *vulnerabilities of the detection models themselves*, particularly the possibility that an intelligent adversary could evade an SVM-based detector. This raises the following question: if an attacker knows that an SVM is being used for detection, can they deliberately craft their signals to avoid triggering alarms? This question has not yet been thoroughly examined in the literature. Existing works have not analyzed how specific feature choices or classifier parameters might be exploited [8], leaving a critical gap regarding the *adversarial resilience of GPS spoofing detection methods*.

In this study, we address the above research gap by investigating the robustness of an SVM-based GPS spoofing detection system against adaptive attackers. We first analyzed the inner workings of the SVM detector to identify a specific weakness in its decision boundary when confronted with cleverly crafted spoofing inputs. Leveraging this insight, we designed a novel GPS spoofing strategy that exploits the identified weakness to evade detection. We then validated the effectiveness of this attack through simulation experiments, demonstrating that a determined adversary can significantly degrade the SVM’s detection capability. The results reveal that an SVM-trained detector, despite its strong performance under normal conditions, can be deceived by an attack tailored to its learned model. This indicates that current spoofing detectors for vehicles are not robust enough against intelligent attacks, suggesting the need for immediate improvements in their adversarial defenses. In summary, the main contributions of this work are as follows:

1. We reveal a critical vulnerability in SVM-based GPS spoofing detection, showing how the classifier’s decision process can be undermined by adversarial inputs.
2. We propose novel GPS spoofing attack strategies that strategically exploit this SVM’s vulnerability to avoid detection.
3. We conducted comprehensive simulations to evaluate the proposed attack against a representative SVM detector, and we demonstrate its success in bypassing the detector. Based on these findings, we discuss the implications for GPS security and outline recommendations for improving spoofing defense mechanisms.

The remainder of this paper is organized as follows. Section 2 provides a background on GPS spoofing attacks, reviews related works, and provides an overview of SVM-based detection and its vulnerability. Section 3 presents the proposed attack strategies and experimental setup. Section 4 reports experimental results that validate the attack’s impact. Section 5 discusses defense-related implications and potential countermeasures to enhance the resilience of GPS spoofing detection. Finally, Section 6 concludes the paper.

## 2. Background and Related Work

### 2.1. GPS Spoofing Attacks and Traditional Countermeasures

**Figure 1:**
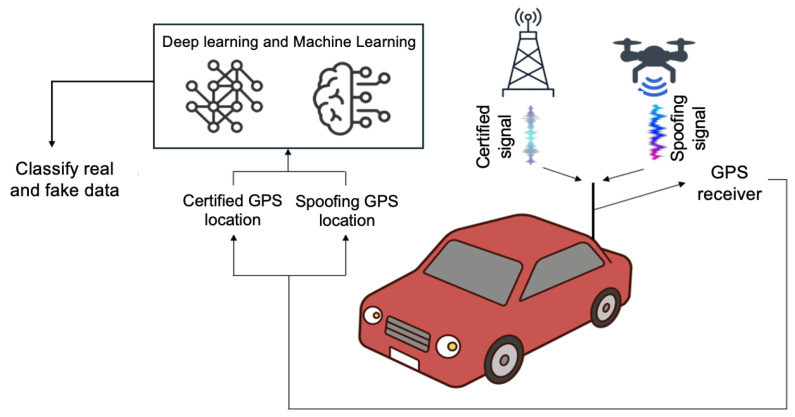
A GPS spoofing attack launched on a vehicle [13].

A GPS spoofing attack refers to an attack where an attacker alters the location information of a vehicle by maliciously manipulating the GPS signal, as shown in Figure 1. Civilian GPS signals are neither encrypted nor authenticated. This inherent insecurity has led researchers to devise many spoofing detection and mitigation techniques. Traditional countermeasures rely on signal processing and hardware techniques to distinguish genuine from fake signals [9,10]. For example, specialized dual-antenna or multi-receiver setups can detect inconsistencies in signal angles or phases, and statistical consistency checks (such as sum-of-squares and difference detectors) can flag anomalous correlations in received satellite signals [11]. Li et al. [12] proposed the Q-channel signal component (QSC), a spoofing detection method that transforms spoofing-induced features into the frequency domain that is then combined with the time domain for a better understanding of signal characteristics. While these classical methods are effective in certain scenarios, they often require additional hardware or fail against sophisticated attackers who precisely emulate genuine signal characteristics. This has motivated the exploration of data-driven approaches using machine learning, which can leverage multiple features of the received signals and receiver state to automatically learn spoofing signatures.

### 2.2. Machine Learning for GPS Spoofing Detection

In recent years, a number of supervised machine learning models have been applied to GPS spoofing detection, showing high accuracy under standard test conditions. Semanjski et al. [14], for instance, developed a support vector machine (SVM) classifier that monitored various GPS signal observables (e.g., signal strength, pseudorange residuals) to detect spoofing; their SVM-based system achieved around 94–96% detection accuracy in experiments with simulated spoofing scenarios. Other researchers have reported even higher performance using advanced models. Shafiee et al. [15] applied a multi-layer perceptron neural network to single-frequency GPS receiver data and achieved over 99% spoofing detection accuracy on a labeled dataset. Tree-based ensemble methods have also been investigated: Aissou et al. [16] evaluated four decision tree ensembles (Random Forest, Gradient Boosting, XGBoost, and LightGBM) for UAV GPS spoofing detection, finding that XGBoost provided the best accuracy (over 95%) with low computational cost. Likewise, deep learning approaches have shown promise. For example, Sun et al. [17] developed a 1D convolutional neural network (CNN) combined with an LSTM to analyze raw GPS signals in real time, and reported detection rates in the high 94–99% range after training on augmented data. Mao et al. [18] proposed a real-time spoofing detection model that learned the temporal patterns of signal quality monitoring (SQM) indicators to determine the presence of spoofing. These studies collectively demonstrate that, under normal (non-adaptive) attack conditions, machine learning models can be very effective at distinguishing spoofed signals from authentic ones, often yielding detection probabilities well above 90% while maintaining low false alarm rates.

### 2.3. Adversarial Machine Learning and Evasion Attacks

However, a critical assumption in the above works is that the spoofing attacks do not adapt intelligently in response to the detection mechanism; in other words, the classifiers are trained and tested on similar attack scenarios, and the attacker is not assumed to deliberately craft their signals to evade the model. This leaves a potential gap: *could a determined adversary fool a high-accuracy GPS spoofing detector by subtly altering the attack?* This question is inspired by the broader field of adversarial machine learning, which has shown that many classifiers (including SVMs and neural networks) are vulnerable to *evasion attacks*. Specifically, researchers have demonstrated that, by making small, carefully optimized perturbations to inputs, an attacker can cause misclassification that still appears normal to human observers or standard metrics. For example, Biggio et al. [19] showed that an SVM-based malware detector could be evaded by perturbing features in a way that exploits the classifier’s decision boundary, and Goodfellow et al. [20] later illustrated how minimal changes to images could mislead deep neural networks (the so-called adversarial examples). Cerracchio et al. [21] evaluated evasion attacks against automotive intrusion detection systems (IDSs), showing that attackers can discover and exploit backdoors to evade detection.

In the context of GPS, an analogous threat is that a spoofer might adjust the counterfeit signals’ parameters to mimic genuine GPS behavior closely enough to avoid detection. Shi et al. [22] demonstrated a generative adversarial network (GAN) that produces spoofed wireless signals which are nearly indistinguishable from genuine signals, significantly reducing detection success rates. In the domain of UAV navigation, Jung et al. [23] focused on adaptive GPS spoofing attacks targeting UAVs equipped with deep-reinforcement-learning-based autonomous navigation systems. They demonstrate that an attacker can inject very small, time-stepped positional deviations that evade internal UAV sensor anomaly detectors, gradually distorting the drone’s trajectory to induce collisions. Alhoraibi et al. [11] recently incorporated adversarial machine learning into a GPS spoofing detection framework, using a GAN to augment training data and improve the detector’s robustness. Notably, their work focuses on strengthening the defense; in contrast, little research to date has focused on systematically analyzing GPS spoofing detection from the attacker’s point of view.

In summary, prior works have established a strong foundation for GPS spoofing detection using machine learning, with many models achieving high accuracy with fixed attack models and datasets. At the same time, studies in adversarial machine learning warn that these models could be fragile against adaptive adversaries. The limitations of the existing literature—such as reliance on non-adaptive or simulated attacks and lack of worst-case analysis—highlight the need for research that evaluates spoofing detection under adversarial conditions. In this study, we address this gap by adopting an attacker’s perspective: we investigate how an intelligent spoofer might exploit the weaknesses of an SVM-based GPS spoofing detector, and analyze the detector’s performance and failure modes in such adversarial scenarios. Our approach sheds light on the robustness of current GPS spoofing defenses and suggests ways to develop stronger detection strategies.

Before presenting our attack methodology, we provide a brief background on SVM classification and outline the known adversarial attack paradigms to frame our approach.

### 2.4. Support Vector Machine: Principles and Vulnerabilities

**Figure 2:**
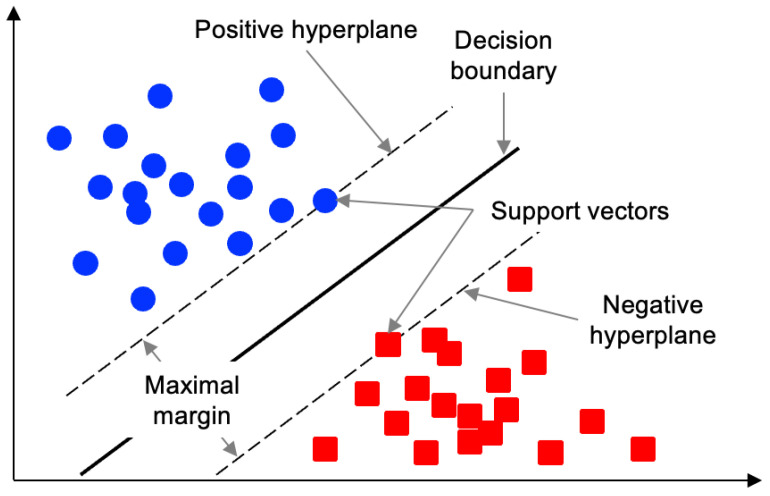
The support vectors define the margin.

Support vector machines (SVMs) are popular supervised learning algorithms for both classification and regression. An SVM learns an optimal decision boundary from training data, which it then uses to predict the categories of new data points [24]. The boundary represents a line that is the criterion for data classification and is also called a separating hyperplane. Because our attack specifically exploits the SVM’s decision boundary, we briefly review how an SVM defines this boundary and margin (Figure 2 shows an SVM hyperplane and support vectors). It is important to find the point where the distance between the red point and the blue point of each class to the hyperplane is maximized, and through this, the most appropriate hyperplane can be defined. At this time, the distance between the hyperplane and the support vector (the data point closest to the hyperplane) is defined as the margin, and maximizing the margin is the key to finding the optimal decision boundary.

Prior studies note several SVM benefits [25]: (1) high accuracy—SVMs often outperform other classifiers in precision; (2) strong generalization—they resist overfitting and handle new data well; and (3) outlier tolerance—SVMs are not overly affected by stray data points. However, SVMs also have drawbacks: (1) scalability issues—training can be slow or memory-intensive on very large datasets; (2) limited interpretability—the model’s decision boundary is not as easily understood as, say, a decision tree; and (3) the curse of dimensionality—with too many features, SVM training can become computationally expensive and performance may degrade.

The SVM classifies the data by determining the optimal hyperplane based on the training data. In this process, the quality of the training data can have a significant impact on classification performance. This study focuses on the sensitivity of the hyperplane, which is vulnerable to intentionally manipulated training data. If an attacker injects incorrect data into the training data, the SVM performs training based on this, and as a result, the accuracy and reliability of the classification result generated by the model can be greatly reduced. These problems can pose a serious threat to the security and reliability of the SVM-based detection model. Therefore, we designed various attack strategies centering on this SVM vulnerability in this study. SVMs have been found to be vulnerable to various types of adversarial attacks, summarized as follows:

1. Evasion attack is a method of deceiving the model by cleverly manipulating input data at the test time. An attacker makes slight modifications to samples that are normally classified as negative, with the goal of causing the SVM to misclassify them as positive. This is implemented by changing the classification result by manipulating a pointer near the decision boundary [26].
2. Poisoning attack is a method of degrading the performance of the model by injecting malicious samples in the training stage. In the case of SVMs, a specially designed malicious sample is inserted into the training data to distort the decision boundary. This can degrade the classification performance of the model [27].
3. Model extraction attack estimates the decision boundary of an SVM to create a replica of the model or identify vulnerabilities. An attacker analyzes the training data to estimate the decision boundary of the SVM and, based on this, predicts the classification of the model or discovers vulnerabilities [28].

In this work, we focus on test-time evasion attacks (Type (1) above), and the other attack types are mentioned for completeness and context. In this study, we designed a *decision boundary attack* strategy that targets the geometric properties of the SVM decision boundary in order to evade detection or cause misclassification. It exploits the mathematical structure of the SVM—especially how it separates classes using a maximal margin hyperplane—to maximize its effectiveness.

## 3. Materials and Methods

### 3.1. Proposed Attack Methods

**Figure 3:**
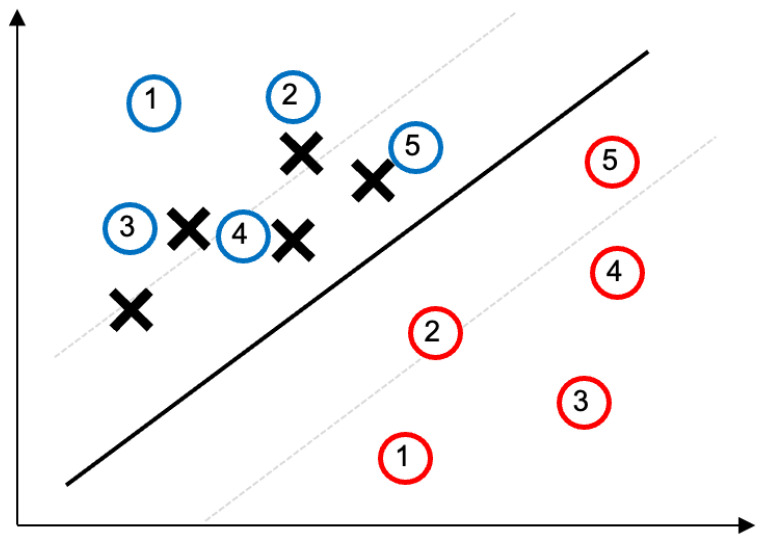
Schematic of the data location shift attack: the circles represents the true path when there is no attack, which defines the decision boundary (solid line) and two hyperplanes (dashed lines). Under the attack, the vehicle’s perceived path (noted as X) gradually diverges from the true path (blue circles) due to incremental GPS offsets.

**Figure 4:**
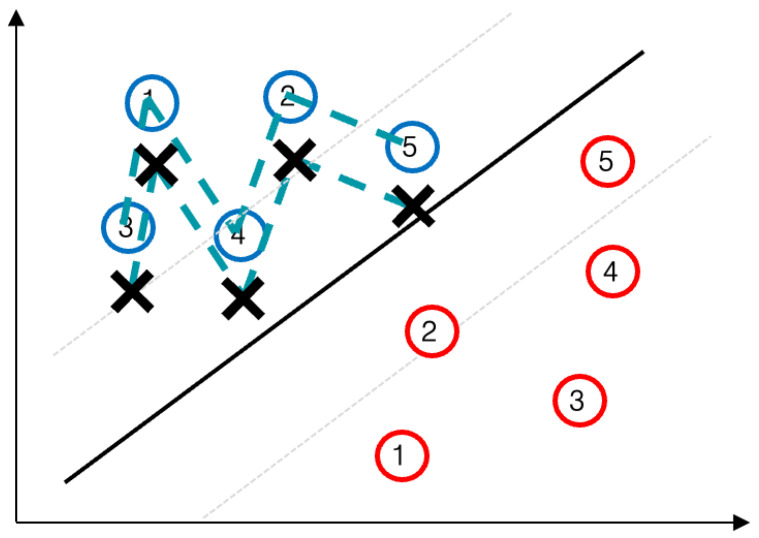
Schematic of the similarity-based noise attack: the overall sequence of small perturbations results in the accumulative noise effect.

This section details the two novel GPS spoofing attack strategies introduced in this work; namely, the *data location shift attack* and the *similarity-based noise attack*. These methods represent the core contribution of our study, as they simulate sophisticated spoofing patterns that could challenge lane-keeping in autonomous vehicles without immediately triggering simple anomaly detectors. More technically, SVMs likely track sudden deviations or certain thresholds but are not triggered by slow or subtle changes. This is how the spoofing patterns are constructed. Therefore, the inherent linear decision boundary of the SVM classifier makes it particularly vulnerable to adversarial GPS spoofing attacks (such as the proposed ones), as these manipulations effectively shift data points across the classifier’s margin, thereby causing misclassification. We also describe the *combined attack*, which integrates both strategies to maximize the spoofing impact. The following subsections explain the concept of each attack, provide step-by-step pseudocode (Algorithms 1 and 2), and illustrate their effects (Figure 3 and Figure 4). This detailed breakdown ensures that readers understand our attack-generation methodology before examining the detection results.
**Algorithm 1** Data location shift attack (DLSA)**Require:**  * *  1:*N*.   Total steps in the scenario  2:$p_{k}=\left(x_{k},y_{k}\right)$.   True GPS position at time step *k*. *x* and *y* represent latitude and longitude, respectively.  3:$\Delta \max=(\Delta x_{\max},\Delta y_{\max})$.   Maximum offset chosen  4:A fixed random seed was used (e.g., np.random.seed(42)).  5:The algorithm runs in O(N) time for *N* time steps.**Ensure:** 
Spoofed GPS output $p_{k}^{\prime }$ for each step k=1 to *N*  6:Δ←(0,0)                       ▹ Initialize offset as zero  7:**for** k=1 to *N* **do**  8:      $\Delta \leftarrow \frac{k}{N}\Delta \max$                 ▹ Incrementally increase the offset  9:      $p_{k}^{\prime }\leftarrow p_{k}+\Delta$              ▹ Apply the current offset to true position10:      Output $p_{k}^{\prime }$ as the spoofed GPS reading at time step *k*11:**end for**

**Algorithm 2** Similarity-based noise attack (SNA)
**Require:**  * *  1:*N*.   Total steps in the scenario  2:$p_{k}=\left(x_{k},y_{k}\right)$.   True GPS position at time step *k*. *x* and *y* represent latitude and longitude, respectively.  3:$\nu_{k}=\left(\epsilon_{k},\zeta_{k}\right)$.   Spoofed noise vector at time step *k*. ϵ and ζ represent latitude and longitude, respectively.  4:*D*.   Distribution modeling normal GPS noise (e.g., N((0,0),Σ))  5:A fixed random seed was used (e.g., np.random.seed(42)).  6:The algorithm runs in O(N) time for *N* time steps.**Ensure:** 
Spoofed GPS output $p_{k}^{\prime }$ for each step k=1 to *N*  7:**for** k=1 to *N* **do**  8:      Sample a noise vector $\nu_{k}=\left(\epsilon_{k},\zeta_{k}\right)∼D$  9:      $p_{k}^{\prime }\leftarrow p_{k}+\nu_{k}$                  ▹ Add noise to the true position10:      Output $p_{k}^{\prime }$ as the spoofed GPS reading at time step *k*11:
**end for**



#### 3.1.1. Data Location Shift Attack

The data location shift attack (DLSA or *shift attack*) involves gradually shifting the reported GPS location of the vehicle over time, causing a slow drift away from the vehicle’s true trajectory. The key idea is to introduce a small positional bias that increases incrementally at each time step rather than a sudden large spoofing jump. By doing so, the attack remains stealthy at any given moment (each individual position change is minor and may fall within normal GPS error margins), but the cumulative effect is that the vehicle’s perceived path is significantly displaced from the real path. Eventually, the vehicle’s control system, tracking the falsified GPS data, guides the vehicle out of its lane. We define a *shift amount* parameter to refer to the maximal distance between the true position of the vehicle and the spoofed position in meters. Thus, a shift amount of 0 implies no attack. In our experiments, we use (−50, −40, …, 0) and note that the minus sign simply shows the direction and that the magnitude is significant.

To formalize the DLSA, let $p_{k}=\left(x_{k},y_{k}\right)$ denote the true position of the vehicle at the *k*-th time step of the simulation (in global coordinates or relative to the map). The attacker defines a gradual offset vector $\Delta_{k}$ [meters, m] that increases with each time step *k*. For example, we can linearly interpolate the offset from zero to a maximum value $\Delta_{\max}$ over *N* steps. At step *k*, the offset is

(1) $$\Delta_{k}=\frac{k}{N}\Delta_{\max}\;,$$

such that $\Delta_{0}=\left(0,0\right)$ (no spoof at start) and $\Delta_{N}=\Delta_{\max}$ (full intended spoof offset at the final step). The spoofed GPS position fed to the vehicle is then given by

(2) $$p_{k}^{\prime }=p_{k}+\Delta_{k}\;.$$

In many cases, the attacker may choose $\Delta_{\max}$, such that it mainly affects the lateral position (perpendicular to the lane direction) while keeping longitudinal errors minimal, since lateral deviation is more likely to cause a lane breach. Algorithm 1 outlines the procedure for executing the data location shift attack step by step. An illustrative example of this attack is shown in Figure 3, where the vehicle’s perceived path (dashed line) steadily drifts away from the true path (solid line) as time progresses.

The circles represents the true path when there is no attack, which eventually defines the decision boundary (solid line) and two hyperplanes (dashed lines). Under the attack,

Each iteration in Algorithm 1 increases the GPS offset slightly. In practice, the rate of increase (slope of the shift) and the direction of $\Delta_{\max}$ can be tuned by the attacker. A slower rate or smaller $\Delta_{\max}$ makes the attack harder to detect over short time spans, whereas a faster or larger shift induces a lane departure sooner (but with higher risk of detection if the shift per step becomes noticeable). This parametric adjustability of DLSA allows attackers to balance stealthiness and effectiveness. By the end of a successful DLSA scenario, the vehicle believes it has stayed on course (since the GPS trail is consistent, only shifted) while, in reality, the vehicle has drifted out of its lane by the distance $|\Delta_{\max}|$.

#### 3.1.2. Similarity-Based Noise Attack

The similarity-based noise attack (SNA or *noise attack*) involves injecting carefully crafted noise into the GPS signal such that each individual perturbation resembles normal GPS noise, yet the overall sequence of perturbations leads the vehicle astray. Under genuine conditions, GPS signals naturally have small random errors, often modeled as zero-mean noise (e.g., Gaussian noise with a certain standard deviation). A naive anomaly detector might ignore such small fluctuations as sensor noise. SNA takes advantage of this by generating a sequence of spoofing noise that statistically mimics the real noise profile (hence, “similarity-based”), ensuring that at no point does the GPS deviation look implausible. However, these perturbations are biased or orchestrated over time to cumulatively cause a significant positioning error, pushing the vehicle out of its lane. We define a *similarity level* parameter to quantify how closely the injected noise resembles genuine GPS noise as a percentage (100% denotes that the noise is indistinguishable from normal noise, and lower values indicate increasingly adversarial noise characteristics). In our experiments, we used discrete similarity levels (0, 20, …, 80) to control noise intensity.

Mathematically, let $\nu_{k}=\left(\epsilon_{k},\zeta_{k}\right)$ denote the attack noise added at time *k*. In a similarity-based noise attack, $\nu_{k}$ is drawn from a distribution *D* that matches the characteristics of genuine GPS error. For instance, *D* can be a Gaussian distribution N((0,0),Σ) with covariance Σ chosen to reflect typical GPS accuracy (e.g., a standard deviation of a few meters). The spoofed position is then

(3) $$p_{k}^{\prime }=p_{k}+\nu_{k},\;\nu_{k}∼D.$$

If *D* is zero-mean, the noise injected at each step has no systematic bias on average, which makes it hard to distinguish from normal noise by simply looking at short time windows. However, the attacker can manipulate the random draws in their favor. One strategy is to bias the noise sequence subtly; for example, over a series of *N* steps, $\nu_{k}$ is drawn such that there is a slight predominance of positive lateral error. Each $\nu_{k}$ remains within a normal range, but the aggregate $\sum_{k=1}^{N}\nu_{k}$ drifts the vehicle gradually in one direction. Another strategy is to occasionally sample a larger deviation that is still within plausible bounds of *D* (e.g., a rare but not impossible GPS glitch), timed in such a way that the vehicle is nudged when it is most vulnerable (such as during a lane change or a curve). The overall effect is a sequence of GPS readings that appear noisy yet benign at a glance, but lead the vehicle off course. Algorithm 2 provides the pseudocode for generating a similarity-based noise attack. Figure 4 illustrates this attack, showing a vehicle trajectory perturbed by noise-like deviations that accumulate into a lane departure over time.

Because SNA relies on random perturbations, it inherently has some uncertainty in its outcomes. Some runs of this attack might result in more drift than others purely due to chance. Nevertheless, as a deliberate attack, SNA can be repeated or tuned until the desired deviation is achieved. Crucially, any defense mechanism that only checks if current GPS noise is within normal variance will be insufficient against SNA since, by design, each $\nu_{k}$ falls within that normal variance. The strength of SNA lies in its subtlety and the attacker’s ability to exploit the stochastic nature of sensor noise.

#### 3.1.3. Combined Attack

We also consider the *combined attack* that integrates both of the above methods to maximize the spoofing efficacy. An adversary in this attack injects a gradual drift offset and superimposes random noise on the GPS signal simultaneously. Formally, the spoofed position under the combined attack at time *k* is

(4) $$p_{k}^{\prime }=p_{k}+\Delta_{k}+\nu_{k},$$

where $\Delta_{k}$ is a slowly growing bias (as in DLSA) and $\nu_{k}$ is a noise term (as in SNA). By combining the two, the attacker benefits from the steady lane displacement caused by the shift attack while using noise to camouflage this displacement. The random jitter can mask the presence of a systematic bias, making it even harder for detection algorithms to isolate the malicious trend. For example, if a defense system estimates and filters out constant biases over time, the noise component can hinder its estimation; conversely, if the system is tolerant to noise, the bias ensures a long-term effect. The combined approach is therefore potentially more dangerous than either attack alone as it merges stealth with effectiveness.

In our experiments, the combined attack was executed by simply applying both algorithms concurrently: at each simulation step, the GPS reading was altered by first adding the incremental shift offset and then adding the noise perturbation for that step. The sequence of spoofed outputs $p_{k}^{\prime }$ thus embodies the characteristics of both the DLSA and the SNA. We observed that this combined method consistently forced the vehicle out of its lane while maintaining a noise profile that looked genuine at each instant. As expected, this scenario posed the greatest challenge for the SVM-based detection system.

### 3.2. Data and Experimental Setup

All experiments were conducted using the CARLA autonomous driving simulator, which is a widely-used, open-source simulator for autonomous driving research built with the Unreal Engine [29]. Its core mechanism is a client–server architecture, where a high-fidelity physics-based simulation runs and an external Python v3.12 or C++ program controls the “ego vehicle”. This allows researchers to test perception, planning, and control algorithms in a realistic environment without a physical car. Its key strengths include realistic simulation with diverse urban maps, dynamic traffic, and a comprehensive suite of sensor models (LiDAR and cameras). Another strength is its flexibility and community support, enabling custom scenarios. However, its main weaknesses are high computational cost and the powerful hardware required for real-time performance, as well as a potential sim-to-real gap, meaning that simulated results may not perfectly translate to the real world.

To ensure the reproducibility and transparency of our experiments, we detail the CARLA simulation setup used to generate GPS trajectories and spoofing attack data. The configurations, consistently applied throughout all experiments, are shown in Table 1. A pre-built urban map (Town10HD_Opt) with marked lanes was used, and a standard sedan model acted as the ego vehicle. The vehicle was set to follow a predefined lane-keeping route under adaptive cruise control conditions, providing a consistent baseline for normal driving (autopilot mode). In the mode, the GPS noise model in CARLA was used after calibrating internal parameters to match the known civilian GNSS error statistics [30]. To generate attack scenarios, we manipulated the vehicle’s GPS data feed in real-time according to our proposed spoofing methods. We defined multiple driving scenarios for data collection: a normal scenario with no GPS spoofing (baseline) and three attack scenarios. In each scenario, the vehicle attempts to drive within its lane; under attack conditions, the spoofed GPS data causes the vehicle to gradually deviate from the lane. This approach allowed us to gather both safe (in-lane) and unsafe (out-of-lane) instances for model training and evaluation. The vehicle’s lateral control in the simulator relies on the (spoofed) GPS position to maintain its lane, making this setup a valid test for a GPS spoofing detector’s effectiveness. We note that the in-lane (1) vs. out-of-lane (0) label can also reflect the observable safety impact of GPS spoofing in a closed-loop control system, while spoofing may be present before lane departure, our labeling scheme focuses on when the spoofing begins to degrade control performance. The controller used is CARLA’s built-in autopilot, which relies on GPS waypoints and uses a PID-based lateral controller. We acknowledge that different controllers may react differently to the same spoofed GPS inputs and that future work could incorporate explicit attack presence labels and time-to-lane-departure metrics to better separate attack detection from safety consequences.

To determine the performance details of the proposed attack methods, we varied the values of several parameters during the experiments. In the *shift attack* scenario, adversarial data were intensively injected into locations selected randomly by an attacker. The shift amount varied from −10 to −50 by 10 in the experiments. In the *noise attack* scenario, adversarial noise was added while maintaining a certain level of similarity to the genuine data. The similarity was set to 0, 20, 40, 60, and 80 in the experiments. In the *combined attack* scenario, two attack methods were applied at the same time with two changing parameters. Using the scenarios, we collected a comprehensive dataset of 10,854 samples. Each sample corresponds to the state of the vehicle at a given simulation time step, including its true position and any applied GPS perturbation from an attack. We assigned a binary label to each sample indicating whether the vehicle was within its lane boundaries (labeled 1) or outside the lane (labeled 0) at that time step. The normal driving scenario produced mostly 1 labels (since the vehicle remained in its lane), whereas the attack scenarios produced sequences of 1 s that eventually turned to 0 once the spoofing caused a lane departure. This labeling strategy reflects the safety outcome: 0 represents a dangerous deviation likely caused by successful GPS spoofing, which is what our detection model aims to predict.

We employed a support vector machine (SVM) classifier as the detection model to distinguish between normal and spoofed conditions. We extracted relevant features from the vehicle’s state and sensor data that could indicate GPS inconsistency or abnormal behavior (e.g., deviations between GPS position and expected lane center position over time). The dataset was randomly divided into a training set (80% of the samples) and a test set (20%). The SVM model was trained on the training set, and hyperparameters (such as the kernel function and regularization parameter *C*) were tuned via cross-validation on the training data. We selected a radial basis function (RBF) kernel for the final model as it provided robust performance on nonlinear patterns in preliminary tests. After training, the model was evaluated on the independent test set. We report standard classification metrics to assess detection performance, including accuracy, precision, recall, F_1_-score, and the area under the receiver operating characteristic curve (ROC-AUC). These metrics provide a comprehensive evaluation of the SVM’s ability to correctly identify spoofing attacks (lane deviations) versus normal driving. Table 2 summarizes the setups for the experiments.

## 4. Results

This section analyzes the detection results and demonstrates how the SVM performed against normal conditions, the individual attacks, and the combined attack.

### 4.1. Accuracy Under Different Attacks

**Figure 5:**
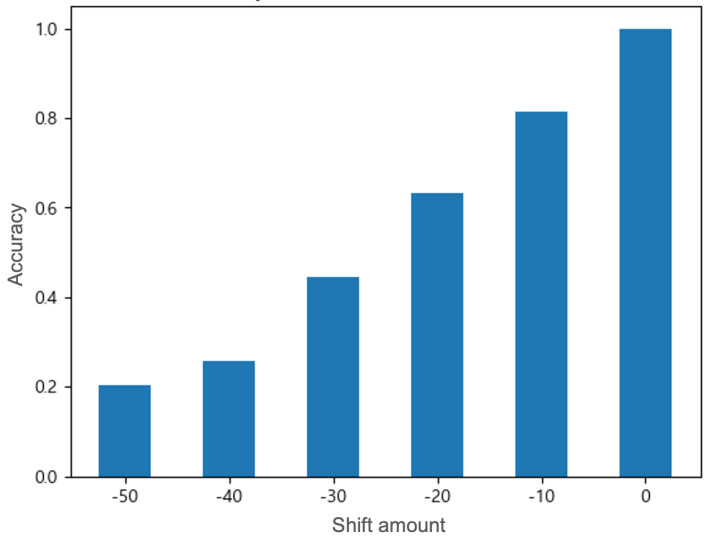
Under the shift attack, the accuracy of the SVM detector gradually decreases as the absolute value of the shift amount [m] increases (from the right side of the x−axis to the left). The average classification accuracy ± standard deviation over 100 runs is reported as follows: 0.999 ± 0.00003, 0.815 ± 0.00029, 0.630 ± 0.00084, 0.445 ± 0.00054, 0.258 ± 0.00046, and 0.204 ± 0.00015.

**Figure 6:**
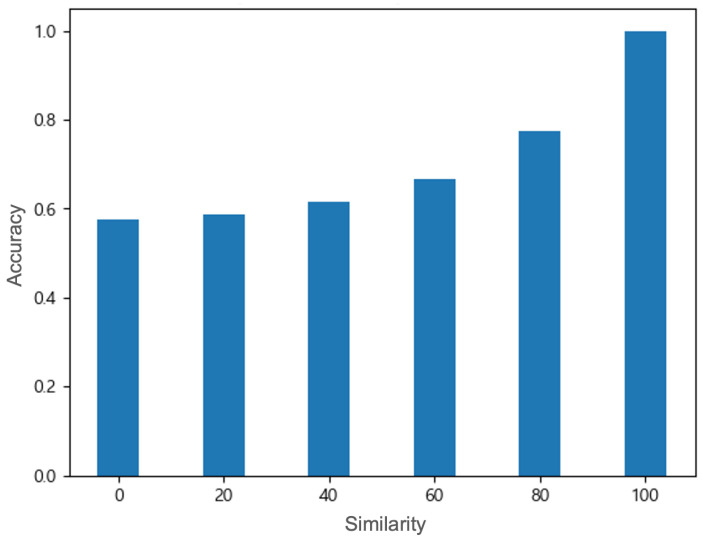
Under the noise attack, the detector’s accuracy sharply decreases as the similarity [%] decreases; from the right side of the x−axis to the left, the average classification accuracy ± standard deviation over 100 independent runs is 0.999 ± 0.00002, 0.774 ± 0.00024, 0.667 ± 0.00045, 0.616 ± 0.00078, 0.588 ± 0.00073, and 0.574 ± 0.00128.

**Figure 7:**
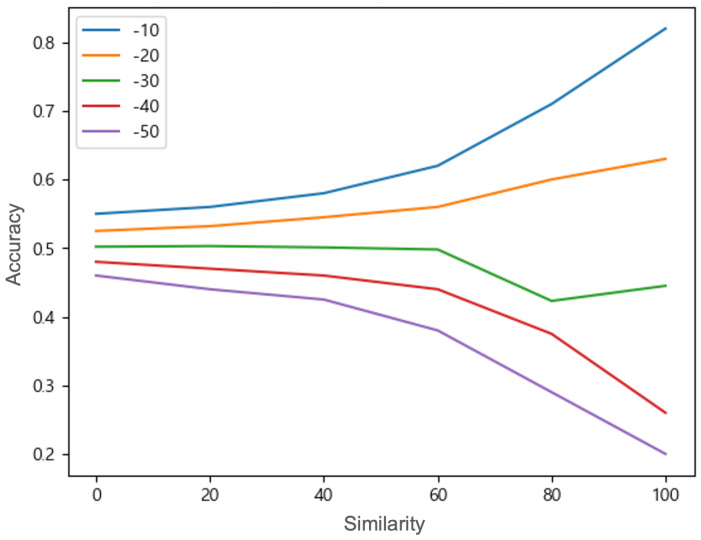
Accuracy under combined attacks exhibits a variety of patterns: a nonlinear interaction between shift and noise changes the threshold for classification dynamically. As the similarity increases, two groups of small shifts (−10 and −20) and large shifts (−40 and −50) change in opposite directions.

Figure 5, Figure 6 and Figure 7 illustrate how the SVM classifier’s overall accuracy varies under the three proposed spoofing attacks (shift, noise, and combined). In the shift attack, the SVM detector shows a high accuracy of more than 99% in the no-attack case (shift amount = 0) (see Figure 5). However, we observe a precipitous accuracy decline: even moderate shift magnitudes cause the classifier’s accuracy to crash sharply (44.5% on −30 and 20.4% on −50). The attack can quickly and effectively degrade the performance of the detector, but it is highly likely to be detected due to rapid changes in the data distribution. In contrast, the noise attack shows a much more gradual degradation: accuracy decreases slowly as the similarity level decreases (from 77.4% on 80 to 57.4% on 0), reflecting that small perturbations have subtler effects (see Figure 6). The attack can degrade the model performance while maintaining similarity to genuine data, so the detection possibility is low. By setting an appropriate level of similarity, effective attacks can be performed while lowering the detection possibility. In summary, the shift attack inflicts the greatest damage on SVM performance, causing near-total failure at high intensities, whereas the noise attack degrades performance more mildly. These findings are consistent with prior adversarial studies: for example, SVM accuracy has been shown to fall dramatically under crafted perturbation conditions (e.g., from 93.5% down to 77.4%) [31]. The rapid accuracy loss under our shift attack indicates that the SVM is highly vulnerable to abrupt GPS data displacements, demonstrating that even relatively small shifts can severely compromise classification accuracy.

Under combined attacks, we observe a nonlinear interaction between shift and noise as shown in Figure 7. At small shift amounts (−10 and −20), adding noise (lowering similarity) makes attacks more effective (accuracy drops further), indicating a synergistic effect. However, at very large shifts (−40 and −50), adding noise actually slightly increases detection difficulty (accuracy improves as similarity decreases) because the shift’s effect dominates. We find there is an intermediate shift ( 30 in magnitude) where increasing noise no longer changes accuracy—the two effects cancel out. This threshold marks a balance point between the two attack factors.

The results demonstrate that the combined attack is not simply a linear combination of the two attacks. Under certain conditions, there is a synergistic effect between the two attack methods, while in other specific conditions, a canceling effect can occur. These results suggest the possibility of more sophisticated attack strategies against SVM models and emphasize the need to develop defense mechanisms against such attacks.

### 4.2. Precision, Recall, and F_1_-Score

Table 3 and Table 4 summarize the detailed detection metrics when changing the values of the shift amount and the similarity. When the shift amount is 0 or the similarity is 100 in both figures, indicating a no-attack scenario, all show high scores. We note that metrics such as the F_1_-score are less informative in scenarios where one class has very few instances. In the no-attack case, essentially all samples are “normal” (negative for attack) and, so, we focus on the trends once attacks are present. Once an attack occurs, the metrics begin to change. We find that as attack strength increases (the shift magnitude increases toward −50, and the similarity decreases toward 0), precision steadily decreases while recall increases. In other words, more attacks yield a higher rate of false alarms (lower precision) and fewer missed detections (higher recall). This trade-off is evident when comparing clean data to attacked data: under spoofing conditions, many normal samples are misclassified as spoofed (raising false positives), whereas more spoofed samples are correctly flagged (reducing false negatives). This pattern matches observations in related intrusion-detection work, where adversarial attacks caused slight drops in precision and F_1_ but improvements in recall [32]. For instance, our strongest noise attacks reduce precision by 46.52%, while recall rises by 20.76%. The net effect is a drop in F_1_-score, reflecting degraded classifier reliability overall.

In summary, stronger spoofing attacks make the SVM more prone to false alarms, indicating that the classifier’s decision boundary is shifted by the perturbations. The precision/recall trends confirm that our attacks systematically inject ambiguity: the SVM becomes over-sensitive (flagging benign data) to malicious inputs. This trend implies that, under attack, the detector would raise many alerts (some false); this could be problematic in practice, but it still misses fewer actual spoofing events than before.

### 4.3. ROC Curve and AUC

**Figure 8:**
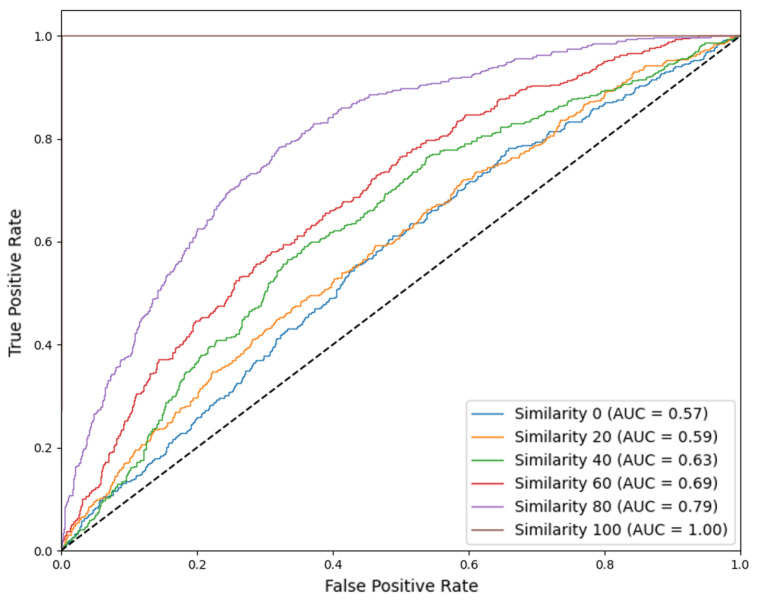
Receiver operating characteristic (ROC) curves for the SVM detector at different similarity levels. In the no-attack scenario (i.e., similarity 100), the AUC achieves 100. As the strength of the attack increases (decreasing similarity), the average value of AUC (over 100 runs) drops to 0.79, 0.69, 0.63, 0.59, and 0.57.

Figure 8 plots the ROC curves for the SVM under varying attack conditions, and Table 5 records the average AUC values and standard deviation. In the no-attack baseline, the ROC curve is near the upper-left corner (AUC ≈ 1.0), indicating excellent separability between genuine and spoofed signals. The ROC curve moves down and to the right as attack intensity grows, shrinking the area under the curve. Quantitatively, we observe a monotonic decrease in AUC: mild attacks (small shift or high similarity noise) yield only a slight drop in AUC ≈ 0.79, whereas strong attacks (large shift or low similarity) can reduce AUC substantially to ≈0.57–0.59. This decline in AUC signifies that the classifier’s ability to distinguish positive from negative samples is weakened. In our context, the AUC drop highlights that our spoofing attacks degrade the SVM’s discriminative power. In particular, the highest-intensity attacks cause the ROC curve to retreat far from the ideal corner, confirming that many spoofed signals become indistinguishable from genuine ones. The deterioration in ROC/AUC with attack strength thus directly demonstrates that our methods compromise the SVM’s detection capability.

### 4.4. Detection Rate

**Figure 9:**
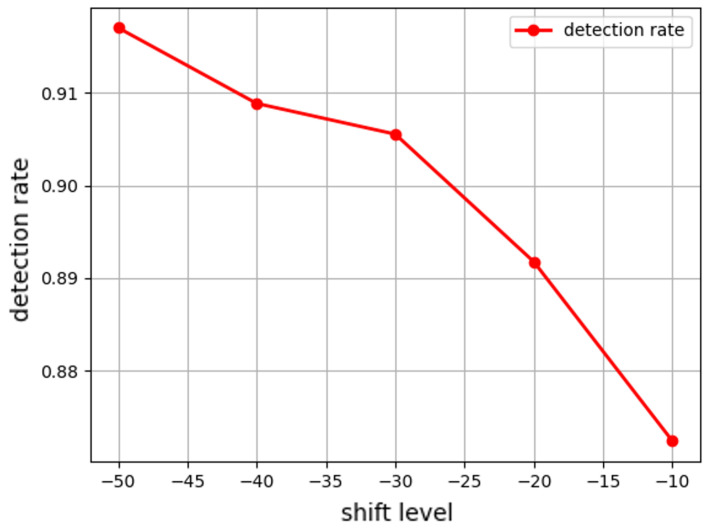
Under the shift attack, as the absolute value of the shift level decreases (from the left side of the x-axis to the right), the attack becomes more subtle and thus less detectable. The average detection rate (over 100 runs) changes from 0.917 to 0.909, 0.905, 0.892, and 0.872.

**Figure 10:**
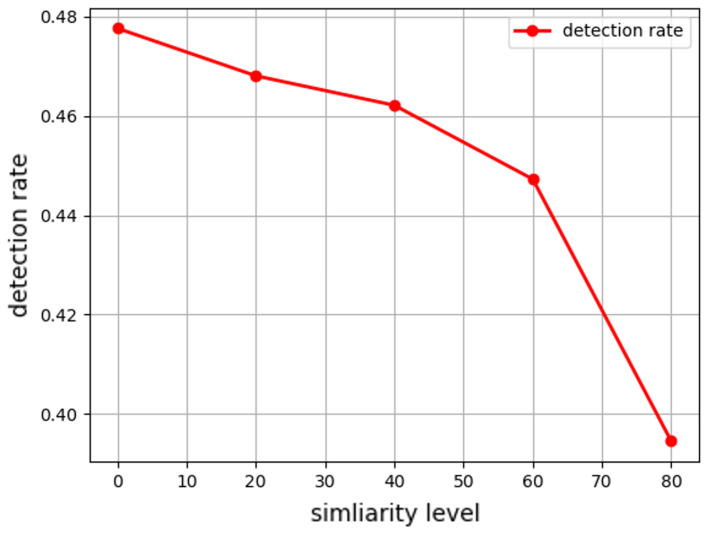
Under the noise attack, as the similarity level increases (from the left side of the x-axis to the right), the spoofing noise becomes similar to civilian GNSS error and thus becomes harder to detect. The average detection rate (over 100 runs) changes from 0.478 to 0.469, 0.462, 0.448, and 0.395.

**Figure 11:**
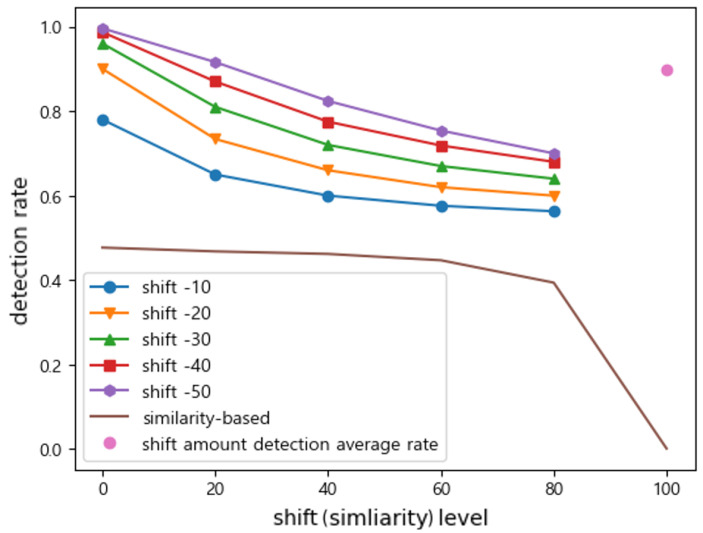
Under the combined attack, the detection rate depends on the balance of shift and noise components. The detection rate is lowered when compared to the shift attack, while we observe a higher detection rate when compared to the noise attack.

Figure 9, Figure 10 and Figure 11 show the detection rate (true positive rate) for each attack as a function of intensity. We note that the settings of shift level = 0 and similarity level = 100 in Figure 9 and Figure 10 represent no-attack scenarios. Since there exists nothing to be detected in the settings, the detection rate becomes always 0; thus, we omit them in the graphs. The shift attack achieves the highest detection rates when the shift is large, as shown in Figure 9. In those cases, nearly all spoofed signals are detected. However, such overt attacks, while easily flagged, drastically affect the model’s performance (as seen by the low accuracy). This indicates that trivial large shifts are not a practical strategy for attackers due to their high detectability. Attempting a new attack that secures the detectability can greatly affect the accuracy of the SVM model, so there is a need to develop a defense mechanism against this.

In contrast, the noise attack yields lower detection rates at comparable intensities. Even when noise is relatively strong (lower similarity), many attacks evade the SVM, making the attack more stealthy (in Figure 10). This provides a better balance between model performance degradation and detection avoidance which results in a more effective attack strategy to reduce the model’s performance while avoiding detection mechanisms. The result emphasizes the importance of developing more sophisticated detection methods that can identify subtle changes in data distribution, especially for attacks that closely mimic data patterns.

The combined attack exhibits intermediate behavior: its detection rate depends on the balance of shift and noise components as shown in Figure 11. Compared to the average detection rate of the shift attack, the detection rate is lowered because the data are shifted with similarity. Compared to that of the noise attack, we observe a higher detection rate when both similarity and shift are varied simultaneously since this results in lower similarity to the genuine data. However, as the absolute value of the shift amount increases, its effect on the decision boundary becomes greater than that of similarity, resulting in higher detection rates.

### 4.5. Detection Rate and Accuracy

**Figure 12:**
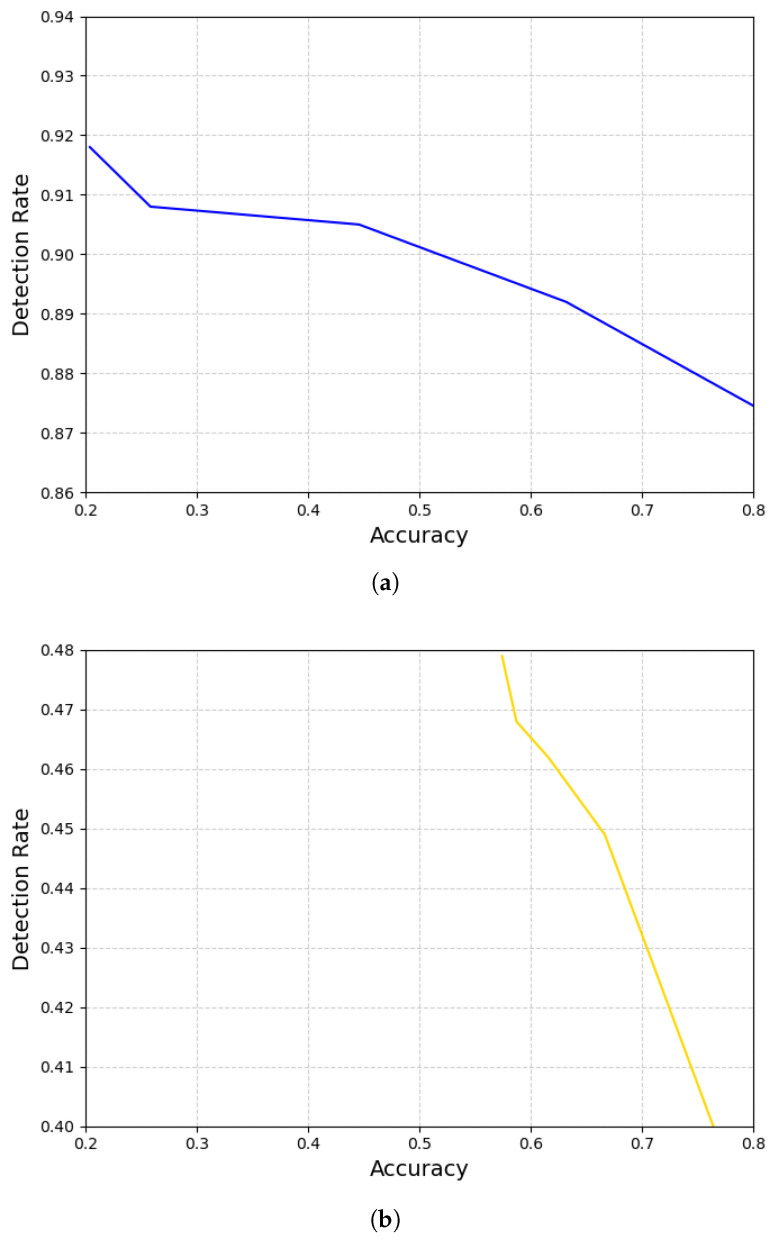
The higher the detection rate, the lower the accuracy. Different attack strategies show different slopes. In the noise attack (**a**), the slope is very similar to that in Figure 9, indicating that the accuracy is almost proportional to the shift amount. However, the slope in the shift attack (**b**) is sharper than that in Figure 10, implying the accuracy is sensitive to the change in the similarity level.

The results consistently show a clear trade-off: overt attacks (large shift) are caught easily (high detection rate) but also severely degrade model performance, whereas subtle attacks (moderate noise) avoid being noticed (low detection rate) while inflicting less obvious damage. These results underscore the balance between the impact and detectability of an attack. Importantly, in all cases, the detection rate never remained high without a corresponding performance drop. Figure 12 confirms their relationship: the higher the detection rate, the lower the accuracy. This is because, as the model becomes more sensitive to adversarial patterns (i.e., when the detection rate is high), it may mistakenly classify some positive samples as negative.

The overall takeaway is that our novel attacks produce substantial distributional disturbance in the GPS data, which the SVM cannot ignore. The high detection for shift attacks (and corresponding accuracy loss) indicates that such perturbations leave a pronounced signature in feature space, while lower detection for noise attacks shows that even small perturbations can slip by if carefully tuned. In sum, every attack scenario demonstrates a significant weakness: the SVM’s detection of spoofing signals is consistently undermined. The quantitative evidence (accuracy, ROC/AUC, precision/recall, detection rate) collectively confirms that the proposed spoofing methods induce serious vulnerability in the SVM model, as evidenced by the dramatic performance declines observed under attack.

## 5. Discussion

### 5.1. Implications and Contributions

Our findings show that an SVM-based GPS spoofing detector works extremely well under some conditions, but can fail considerably under others. On the one hand, the SVM classifier demonstrated excellent performance under standard (non-adversarial) conditions, achieving near-perfect spoofing detection accuracy in our simulations. This result reaffirms that even relatively lightweight machine learning models can significantly enhance navigation security by flagging spoofing attempts in nominal scenarios. On the other hand, our study revealed that these strengths can become liabilities in the face of an adaptive attacker. The fixed decision boundary learned by the model was systematically exploited by subtle, carefully crafted inputs, leading to drastic drops in detection efficacy. In particular, gradually shifting the vehicle’s reported location or injecting noise that mimics normal GPS jitter can deceive the SVM-based detector for a prolonged period, even as the vehicle deviates off course. This dichotomy highlights that high accuracy on static test sets does not guarantee robustness in adversarial settings, implying that traditional evaluation metrics may paint an overly optimistic picture of security unless adversarial resilience is also assessed.

This study makes several noteworthy contributions to the field of GNSS security and machine-learning-based intrusion detection. First, we empirically demonstrate that a GPS spoofing detection system using an SVM—which performs well under normal conditions—can be systematically undermined by an intelligent adversary. We identified and analyzed a specific weakness in the classifier’s decision mechanism, showing how adversarially crafted inputs (e.g., incremental location shifts and similarity-mimicking noise) exploit the SVM’s assumption of a static decision boundary by operating near that boundary to evade detection. Second, we designed and implemented two novel attack strategies (the data location shift attack and the similarity-based noise attack), along with their combination, to illustrate practical evasion techniques against the SVM. Through extensive simulations, we showed that these adaptive attacks induce significant degradation in detection metrics (such as accuracy and true positive rate), thereby quantifying the risk posed by adversarial spoofing. Third, based on these findings, we outline recommendations for improving spoofing defenses, such as real-world evaluations, detection algorithm hardening, and integration with additional sensor inputs. By pinpointing where the SVM approach fails, our work helps to direct the development of more robust countermeasures. Overall, our work improves what is known about GPS spoofing detection when attackers adapt. It also links the ideal performance of our detector (in lab settings) with how it actually fares against real adversaries.

### 5.2. Limitations

Several limitations must be acknowledged. First, our experimental validation was conducted exclusively in the CARLA simulation environment, which may not capture all complexities of real-world GPS signal propagation and spoofing scenarios. The controlled nature of simulation environments limits the generalizability of attack effectiveness to actual autonomous vehicles operating under diverse environmental conditions and with varying hardware configurations. Second, our attack model assumes perfect knowledge of the target SVM’s decision boundary, representing a white-box scenario. Real-world attackers would likely face black-box conditions with limited knowledge of detection system parameters, while adversarial transferability suggests attacks developed against one model can often succeed against others, the effectiveness of our specific techniques under black-box conditions requires further investigation. Third, our dataset of 10,854 samples, while comprehensive, was generated from specific driving scenarios and may not represent the full diversity of autonomous vehicle operations across different geographical regions, weather conditions, or traffic patterns. The binary classification approach (in-lane vs. out-of-lane) also simplifies the complex safety assessment requirements of actual autonomous driving systems. Next, our study centered on an SVM classifier; other detection models (e.g., deep neural networks or ensemble methods) might exhibit different or additional vulnerabilities. Evaluating those was beyond our scope. Lastly, we note that we did not implement a specific defense mechanism in this work, as our focus was on exposing the vulnerability; designing and testing effective countermeasures is left for future research. Despite these limitations, the demonstrated attacks clearly reveal an Achilles’ heel in the detection approach under ideal attacker assumptions. Real-world testing will be needed to fully quantify the associated risk, but our results serve as an early warning.

### 5.3. Future Directions

Building on this research, future work should address the above limitations and explore enhancements to the GPS spoofing detection framework. A primary next step is to validate the SVM-based detector and the identified vulnerabilities in real-world settings. This includes field trials with actual vehicles and controlled spoofing signals, in order to confirm whether the attacks remain effective outside the simulator and to observe the detector’s performance under genuine operating conditions. Such in situ evaluations are crucial for translating our findings into practical security recommendations. Furthermore, evaluating other machine learning models (e.g., deep neural networks or decision tree ensembles) with similar adaptive attacks would be valuable as their decision surfaces may differ from those of SVMs. Whether such models offer greater robustness or merely different vulnerabilities is an open question. Another important direction is to develop countermeasures that improve the adversarial robustness of GPS spoofing detection. One approach is to harden the detection algorithm itself. For instance, adaptive or more complex classifiers that account for temporal patterns in the data might better recognize slowly evolving attacks. Incorporating techniques from adversarial machine learning (say, adversarial training) could further strengthen the SVM or similar classifiers against the demonstrated exploits. Integrating additional sensor inputs can further strengthen spoofing detection. Modern vehicles are equipped with inertial measurement units (IMUs), odometry sensors, cameras, and digital maps. Cross-validating GPS readings against these independent sources—for instance, comparing the GPS-derived position with IMU-based dead-reckoning or with lane information from cameras—could help to detect inconsistencies caused by spoofing. Going forward, we recommend strengthening GPS spoofing defense by combining better algorithms, adversarially trained models, and cross-checks with other sensors. Taking these steps could make autonomous vehicle navigation more secure and help to protect against the constantly evolving tactics of attackers.

## 6. Conclusions

In conclusion, this study revealed a critical vulnerability in SVM-based GPS spoofing detection for autonomous vehicles. We designed a series of adaptive spoofing attacks (the data location shift and similarity-based noise attacks) that exploit weaknesses in the SVM’s decision boundary to evade detection. The simulation results showed that even a high-performance SVM detector can be deceived by such adversarial inputs, leading to a significant drop in detection accuracy. This is an invaluable demonstration that an SVM-based GPS spoofing detector can be systematically evaded by an adaptive attack, revealing a realistic threat to autonomous vehicle navigation security. Therefore, it is essential to advance our detection and defense mechanisms to recognize these attacks, in order to ensure the safe and reliable use of GPS in autonomous vehicles. We hope this study spurs further research into adversarially robust GNSS spoofing defenses, ultimately helping to safeguard autonomous vehicles against evolving cyber–physical threats.

## Figures and Tables

**Table 1:** Configurations for CARLA autonomous driving simulation.

**CARLA Version**	0.9.15
**Map Environment**	Town10HD_Opt
**Vehicle Model**	Lincoln MKZ 2020 (default CARLA ego-vehicle)
**GPS Sensor**	Carla.GnssSensor (update rate = 1 Hz)
**Data Logging**	Position and lane offset logged at each time step
**Control Agent**	CARLA Autopilot API

**Table 2:** Parameters and configurations used for experiments.

Parameter	Setup
**Dataset**	GPS coordinates from the CARLA driving simulation
**Number of samples**	10,854 samples in total - 80% of samples for training and 20% for testing - 80% of samples from attack class and 20% from no-attack class.
**Number of runs**	100 runs over multiple random seeds
**Spoofing detection model**	SVM with RBF (radial basis function) kernel, linear hyperplane, regularization parameter C = 1.0, gamma = scale
**Attack scenarios**	Shift attack, noise attack, combined attack
**Maximum offset for Shift**	(−50, −40, …, 0)
**GPS noise model**	Gaussian noise, $N(0,\sigma^{2})$ with calibration (noise_lat_stddev = 0.000015 deg and noise_lon_stddev = 0.00002 deg)
**Noise scaling factor**	(0, 20, …, 80)
**Baseline scenario**	No GPS spoofing with autopilot mode

**Table 3:** Classification metrics and confusion matrices for varying shift amounts. A shift amount of 0 represents a no-attack scenario, while −50 denotes the strongest shift attack.

Shift Amount	Precision	Recall	F_1_-Score	TP	FP	FN	TN
0	0.999	0.997	0.998	2204	2	7	8641
−10	0.522	0.875	0.654	1935	1772	276	6871
−20	0.355	0.900	0.510	1990	3615	221	5028
−30	0.269	0.906	0.415	2003	5444	208	3199
−40	0.216	0.909	0.350	2010	7295	201	1348
−50	0.204	0.919	0.333	2032	7928	179	715

**Table 4:** Classification metrics and confusion matrices for varying similarity levels. A similarity of 100 represents a no-attack scenario, while 0 denotes the strongest noise attack.

Similarity Level	Precision	Recall	F_1_-Score	TP	FP	FN	TN
100	0.998	0.999	0.999	2209	4	2	8639
80	0.460	0.395	0.523	873	1025	1338	7618
60	0.319	0.447	0.404	988	2110	1223	6533
40	0.274	0.462	0.364	1021	2707	1190	5936
20	0.254	0.468	0.344	1035	3039	1176	5604
0	0.246	0.477	0.334	1055	3233	1156	5410

**Table 5:** Average and standard deviation of AUC values across similarity levels.

Similarity Level	Average AUC	Standard Deviation
0	0.574	0.0158
20	0.593	0.0225
40	0.630	0.0100
60	0.688	0.0147
80	0.793	0.0094
100	1.000	0.0000

## Data Availability

This research is driven by the urgent need to understand and mitigate emerging threats in autonomous vehicle navigation systems, specifically those involving adversarial manipulation of GNSS signals. All experiments were conducted in simulation environments (e.g., CARLA) without any real-world signal interference. No field transmission, RF equipment, or physical spoofing was performed. To prevent misuse, we do not release raw attack generation code or replay-capable GNSS traces. Any shared material (e.g., defense modules or sanitized data) is limited to supporting anomaly detection and mitigation research. We follow responsible disclosure principles and welcome collaborations on secure-by-design vehicular systems.
